# A fully homomorphic encryption federated learning architecture for privacy preserving in industrial internet of things

**DOI:** 10.1016/j.mex.2026.103898

**Published:** 2026-04-03

**Authors:** Shraddha Subhedar, Deepa Parasar

**Affiliations:** Amity School of Engineering and Technology, Amity University Maharashtra, Mumbai 410206, India

**Keywords:** Cheon-Kim-Kim-Song encryption, Non-IID, Global aggregation, Deep learning

## Abstract

We are currently entering the fifth revolution of industry - Industry 5.0. IIoT is the domain where massive quantities of data are flourished by the associated devices in an industry on a daily basis. To realize industry 4.0, the Industrial internet of things is considered a prominent one. Federated Learning, also known as collaborative learning, employs a decentralized approach in its applicability while maintaining data privacy, but many existing frameworks struggle with handling privacy of gradients which are transferred to Federated servers. Unlike conventional approaches, proposed fully homomorphic encryption based Federated Learning-FHEEFL ensures that raw gradients never leave local IoT nodes; instead, only updates or changes in model secured with encryption techniques are transmitted. The framework achieves a very strong privacy as well as data security. FHEEFL is intended for lightweight to moderate-capacity models characteristically labouring in Edge-IIoTset analytics, where privacy guarantees must be well-adjusted with computational feasibility. While CKKS-based encrypted aggregation incurs additional overhead, the framework establishes practical applicability for privacy-critical industrial tasks under realistic resource constraints. It is proven as a privacy-centric federated learning solution, setting a new benchmark in tackling key challenges in data security and privacy. The proposed method is implemented using Edge-IIoTset dataset. The proposed fully homomorphic encryption based Federated Learning-FHEEFL method is tested in IIOT scenarios.

• FHEEFL method provides better privacy with better memory usage, CPU usage and throughput parameters.
• Performance is analysed with 4 variations of models- Tiny, small, medium and large.

## Graphical abstract

**Figure:**
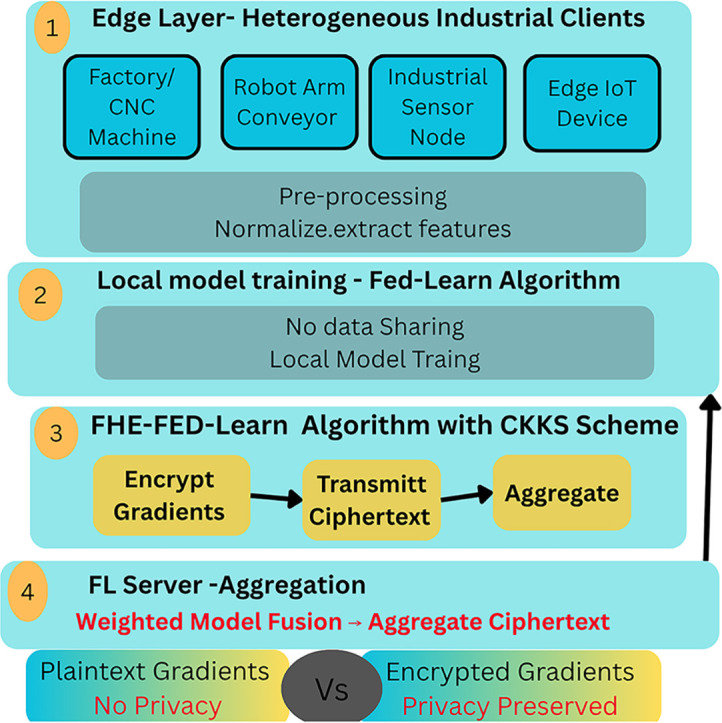

**Table:** Specifications table.

**Subject area**	Engineering
**More specific subject area**	Federated learning model applicable in Industrial Internet of thing
**Name of your method**	FHEEFL: Fully Homomorphic Encryption Enabled Federated Learning
**Name and reference of original method**	None
**Resource availability**	None

## Background

IIoT is a huge network that consists of different types of physical objects and virtual objects. All objects connect and share data collected from adjacent environments. With the employment of intelligent devices, supplementary conditions for the forming of a sharing model are created. Traditionally, Industry data was of very small quantity whereas nowadays we are dealing with huge data silos on a daily basis [1]. In the old days data used to be transferred was very simple with no many dependencies. Today data generated itself has a very complex structure. Thirdly, in the previous Industry environment, data transmission was unidirectional whereas now in the era of edge computing, two-way data transmission is common [2]. So, privacy and security requirements in IIoT are crucial now which ultimately motivates us to contribute to this domain.

Proposed work mainly exploits the functionality of Federated learning in IIoT. Federated Learning is a classical approach which provides a complete decentralized approach. FL has a special capability of collecting global knowledge from all the distributed nodes. Here, the significant benefit of Federated learning is that clients involved in the environment never share their raw data with anyone. So, the privacy of client nodes is always preserved. The application domain chosen for our experimentation in IIoT. It is the domain where massive quantities of data are induced by the associated devices in an industry on a daily basis. This is why, for the implementation of Industry 4.0, IIoT is considered as one of the pivotal technologies. The generalized objective can be to enhance the efficiency of IIOT network by integrating advanced techniques along with it. Nowadays we are dealing with huge data silos with a very complex structure on a daily basis in the IIoT domain. It is justified to say that improving efficiency with changing scenarios is the need of the hour. Hence, a concurrent Fully homomorphic encryption enabled federated learning (FHEEFL) framework is built by applying encryption schemes while training the local model and securing gradients located on edge hosts. We coin the approach as FHEEFL. In the proposed methodology, an untrusted server will be strictly prohibited to check the private data shared from edge hosts as the outputs shared with the server will be in encrypted form. Thus, we have achieved nullification of data reconstruction as well as model inversion attack. Moreover, the concurrency within our framework guarantees good performance when compared to common Machine learning /deep learning techniques.

## Contribution

1) This method document presents an experiential training method for IIoT computing scenarios with Fully privacy preservation.
2) We introduce the concept of concurrency to the IIoT environment incorporated with federated learning, where cooperative decision making takes place among the edge nodes and the server. Moreover, the need of sharing complete models can be avoided by sharing only the outputs in the form of gradients with secure encryption. Here, an identical algorithm can be utilized in other collaborative scenarios, where lightweight to medium-scale models are deployed.

## Method details

Federated learning is the new imperative to deal with data privacy challenges in decentralized systems. The important breakthroughs that this section reviews, along with their relevant connections, are of immense value to the creation and building of the FHEEFL framework.

In the paper [3] entitled “SAFE: A Scalable Homomorphic Encryption Accelerator for Vertical Federated Learning”, the crucial need for privacy preservation in scenarios involving cross-agency data cooperation, particularly in Vertical Federated Learning (VFL) is addressed. While VFL allows multiple parties with different features but shared users to train a model together without exchanging raw data, the interaction and exchange of information during the training process can still expose sensitive data. The SAFE framework is proposed as an HE accelerator specifically designed for scalable homomorphic matrix-vector products (HMVPs), which are identified as the primary performance bottleneck in VFL training applications like logistic regression. The core idea is to accelerate this specific, performance-limiting operation to make HE-based VFL more practical. The hardware architecture is customized for the HMVP dataflow, supporting both spatial and temporal parallelization of functional units. In summary, SAFE is an FPGA-based hardware accelerator designed to overcome the performance bottleneck of Homomorphic Matrix-Vector Products in HE-based Vertical Federated Learning, thereby enabling practical and privacy-preserving cross-agency data cooperation and model training. In the same year, Jiahui Wu and Weizhe Zhang [4] proposed a FHE variant form named ESAFL. This algorithm aimed for Cross-Silo Federated Learning by employing Additive Homomorphic Encryption. This scheme provides a methodical and systematic approach to cross-silo based federated learning offering strong privacy guarantees, particularly against untrusted clients and third parties, while significantly improving computational and communication efficiency compared to prior, HE-based methods. It utilizes an encryption algorithm built upon the hard problem of RLWE. The ring learning with errors problem underpins cryptographic algorithms that are resistant to quantum attacks and also forms the basis for homomorphic encryption methods like BFV [5] and CKKS [6]. Wei-Tao Song et al. [7] has proposed a scheme, called ILP-FHE, to address the challenge of storage complexities in existing FHE schemes, particularly for applications in the Internet of Things (IoT). The paper discussed two contributions: A Key Switching Technology with minimum noise and A Bootstrapping-Less levelled FHE. Key switching used is based on a novel multiband decomposition to achieve better noise control. The scheme utilizes the Bin-LWE assumption twice. A levelled FHE scheme can evaluate circuits up to a predefined depth without requiring the costly bootstrapping procedure. It has proven performance benefits over the Bra scheme [8] in terms of parameters, key/ciphertext sizes, and computation costs. In 2022, Yange Chen et al. [9] addressed the hypercritical issues in Federated Learning (FL) such as security and privacy when applied to Industrial Internet of Things (IIoT) environments. Notably, Yange Chen et. al. designed a scheme called DeepPAR, an asynchronously operating method utilizing deep learning for privacy-preservation by incorporating proxy re-encryption. This paper, however, performs an analysis and improvisation of the DeepPAR scheme proposed by Zhang et al. [10] and identifies significant security vulnerabilities. The authors prove that the original DeepPAR scheme has vulnerabilities in its process for generating re-encryption keys, which may result in the exposure of secret keys owned by either participants or the proxy server1. Additionally, it is shown that the original scheme is susceptible to collusion attacks involving the parameter server and participants. ElGamal Cryptography is used as an encryption scheme. It is a better and more secure scheme in privacy-preserving scenarios in IIoT. FHE serves as a prominent solution to ensure data privacy by enabling computations directly on encrypted data. However, its notorious performance degradation severely limits the practical application, due to the explosion of both the ciphertext volume and computation.

Zehao Chen et. al [11] proposed a robust acceleration framework named as HMC-FHE used in a heterogeneous environment at the data processing sites employing Homomorphic Encryption. This architecture combines a centralized GPU, designed for computation-intensive tasks, with multiple auxiliary Hybrid Memory Cube (HMC) processing engines, which are well-suited for memory-intensive workloads due to their high internal bandwidth and low latency. The GPU and HMCs are connected directly via memory links, and a CPU oversees task scheduling and resource management. HMC-FHE incorporates four key hardware/software co-design techniques- Fine-grained kernel offloading mechanism, Bandwidth-Aware Ciphertext Partitioning (BaCP), Kernel Execution Pipeline and Kernel Tuning Scheme. Evaluations were conducted using a simulated GPU-HMC environment integrating GPGPU-Sim and CasHMC simulators. The framework was tested on six neural network models relevant to privacy-preserving machine learning (CNN, LeNet, St-GCN, AlexNet, VGG-16, and ResNet-20).

Privacy preservation is the primary feature of FL as raw data never leaves the local node. Decentralization is employed as the training data is distributed across multiple devices or clients. Devices connected to the network can participate in training asynchronously i.e. with no worry of global clock synchronization.

**Fig. 1:**
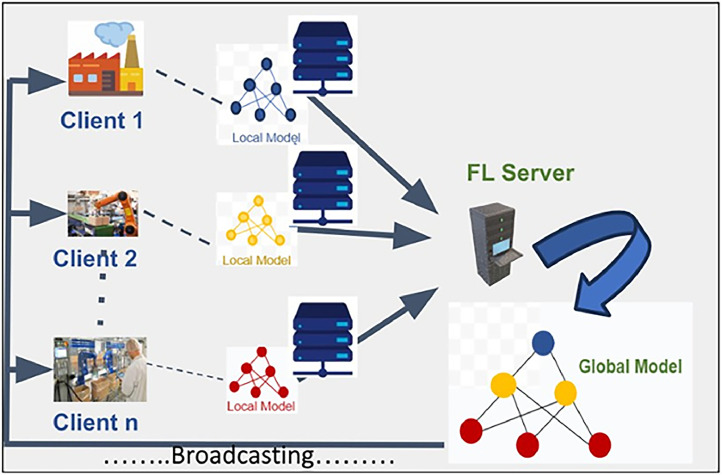
Federated Learning in IIoT.

**Fig. 2:**
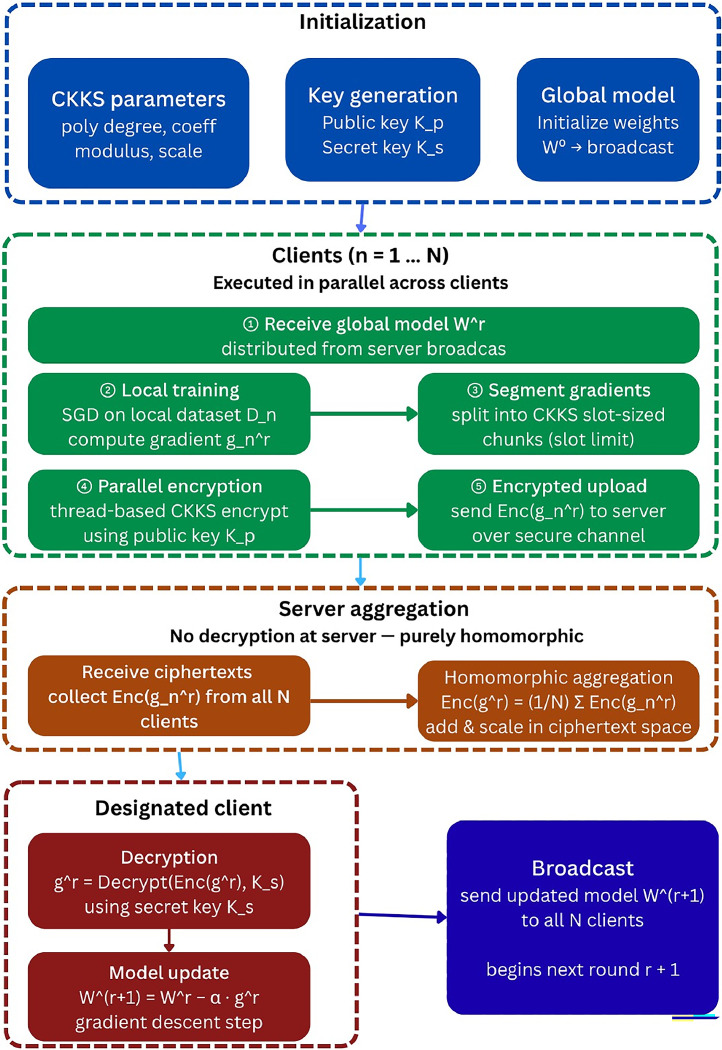
Architectural Workflow of FHEEFL.

Fig. 1 shows an idea of incorporating FL in the domain of IIoT. Data handled here will be non-IID (non-independent and identically distributed). This data refers to datasets where data points are not both independent and drawn from the same probability distribution. In simpler terms, each data point doesn't have the same chance of being selected, and their values might be related to each other. This contrasts with IID data, where each data point is assumed to be independent and drawn from the same distribution. As in federated learning, data is distributed across multiple devices, each device might have a unique distribution of data, so it produces NON-IID data. The model training workflow using federated learning concept is depicted in Fig. 2. Three parties are involved in the IIoT scenario based on FHEEFL, including a server and participating client nodes and respective edge nodes.

The role played by different entities are as follows:

1) Federated Server: This is the core component of the framework which has dominant storage capacity and computational power. The Federated server conducts an averaging process by gathering the ciphered gradients. It then updates the global model and communicates to the all-participating nodes with transmission of global weight changes.
2) Edge nodes: Edge nodes are computing resources located at the network's edge, closer to the data source (i. e. Local Client nodes) than a federated server. These edge devices gather information from sensors or IoT devices, creating their own exclusive data sets. They collaborate to enhance the accuracy of a model. Each device independently trains the model using its own data set to generate a fresh set of gradients. These gradients are then encrypted with their private keys and transmitted to the federated server.
3) Local Client nodes: Nodes are the fundamental building blocks of an IIoT system, representing the physical devices that interact with the real world. They are responsible for collecting data through various devices such as Sensors (temperature, pressure, vibration), cameras, RFID readers, actuators etc. Client Nodes connect to the network, through edge nodes, to transmit their data.

As depicted in Fig. 2, the FL server node collaborates with N client nodes to establish a FL network group. Each client-n (where n ranges from 1 to N) possesses its own dataset D_n, which remains private and is not shared with other nodes acting as clients. The FL Aggregator harmonizes all N nodes in global model development which seeks continuously in loss function reduction globally. This model learning process can be illustrated using stochastic gradient descent (SGD).

### Local learning and upload

In case of each client-*n* (where n ranges from 1 to N), local model (W_n_, r) is trained using the data collected at the client locally. This is done with the utmost care to keep the loss function *L* least as possible and learning rate α will be employed to train the model. For each iteration r, weights of the nth client will be updated using the following mathematical equation. This update will be done by the SGD component shown in Algorithm 1.

(1) (Wn,r)=(Wn,r−1)−α∇wL(Wn,r−1)

**Algorithm 1:** Fed_Learn.

**Input:** Local Dataset D_n, W^0^
**Output:** Updates to Global model
1. Initialize global model W^0^
2. For each global round r = 1 to R
3. Each client n ą {1, ……, N}
4. Receive global model W^r^ from server
5. Train model on local dataset D_n
6. Compute gradient g_n^r^ using SGD
7. Send g_n^r^ to server
8.Server:
9. Aggregate gradients:
10. G^r^ = (1/N) * ∑g – n^r^
11.Update global model:
12. W ^(r+1)^ = W^r^ - ă * g^r^

The (Wn, r) is uploaded to the server.

### Global aggregation

The weights (Wn,r) calculated at each client in the previous step- Local learning and upload, are gathered by the server at each iteration and aggregation is performed. These outcomes the global model *W* which can be represented as follows:

(2) $$Wr\;=\frac{\;\sum_{n=1}^{N}\left|Dn\right|Wn,\;r}{\sum_{n=1}^{N}\left|Dn\right|}n=1,2,\ldots ..N\;\;$$

Server will decide the contribution of each client *n* in proportion to the dataset D_n which is generated locally at the same site where client n is resided. Then averaging will take place at the server to constitute the global model. Then these updates are communicated at the node sites *N*. Then the process of global model updating occurs. Here, the required action is the calculation of loss function which is performed locally by each of the client *n* by utilizing dataset D_n. This step is performed in an iterative and distributed manner, which prominently helps to refine and enhance the global model state after training with each update. This intricacy of FL in distributed scenarios leads to minimization loss function globally across all clients' datasets. leading to overall improvement of the method.

Algorithm 1 shows the step wise flow of the technique.

### Fully homomorphic encryption

It is an extension of LHE (Levelled Homomorphic Encryption) with bootstrapping. This HE types supports the evaluation of arbitrary circuits by granting unlimited addition and multiplication. The robustness of FHE is attributed to this mechanism; it limits the noise level within a ciphertext, thereby permitting more computations on the data [12]. However, bootstrapping is a highly expensive technique, being more than an order of magnitude bit slower than other HE operations.

The “privacy homomorphism” is a concept from which the notion of fully homomorphic encryption (FHE) derived. Rivest et al. proposed this notion very first [13]. More formally, we define this homomorphism as:

(3) Enc(x⊙y)=Enc(x)⊙Enc(y)

where Enc is Public Key encryption, stands for addition or multiplication. x and y are plaintext.

Especially, in demanding scenarios like cloud computing and data outsourcing, the profound association of FHE is observed in addressing privacy and security concerns across varied applications. FHE is proven as a profound solution in an untrusted environment, crucially solving the confidentiality issues, primarily when a third party is involved in the scenarios. Employing FHE, it becomes easier to entrust data and operations in realizing secure computing in an untrusted environment [14,15]. With FHE, as computations are only permitted on encrypted data, neither resultant calculations nor data itself is exposed to a third party.

### Proposed FHEEFL framework

We have proposed a system which is hosted on a Federated framework enabled with Fully homomorphic encryption. The main objective is to provide privacy to the crucial data generated over the IIOT network. While numerous privacy-preserving federated learning methods have been introduced, many depend on either additive-only homomorphic encryption or the injection of statistical noise. Additive schemes restrict encrypted computations to aggregation tasks and might necessitate protocol-level changes for weighted updates, whereas differential privacy involves a trade-off between accuracy and privacy. Secure multi-party computation methods, though reliable, often require several communication rounds and synchronization, leading to overhead. In contrast, the proposed framework utilizes CKKS-based fully homomorphic encryption to facilitate encrypted weighted aggregation without the need for intermediate decryption, thus minimizing trust requirements while preserving training accuracy. This elevates the contribution from mere architectural integration to a methodologically unique privacy-preserving aggregation approach. FHE provides multiple variants such as CKKS (Cheon-Kim-Kim-Song) [16], Brakerski-Gentry-Vaikuntanathan (BGV) [8], and the Brakerski/Fan-Vercauteren scheme (BFV). In abovementioned schemes, CKKS has gained high acceptance owing to its adeptness in handling real numbers. Analogous to public key encryption (PKE), the Cheon scheme encompasses techniques of encryption, decryption and key generation. Nevertheless, distinct from PKE, homomorphic addition and multiplication operations are amalgamated by CKKS enabling secure addition or multiplication of two ciphertexts [17]. CKKS supports operations like addition and multiplication on encrypted vectors, which are critical for aggregating model weights in FL [18]. In RLWE-based HE schemes, the plaintext domain is typically a cyclotomic polynomial ring Zq[X] / (Xn + 1) with a finite characteristic. But the messages that require encryption in federated learning are real numbers. Consequently, it is necessary to encode these real numbers as integral coefficient plaintexts within the ring Zq[X] / (Xn + 1) prior to encryption [19]. We have used polynomial arithmetic to strike a balance among computational adeptness and strong security. A method for efficiently encoding and decoding between complex vectors and integral coefficient polynomials has been employed. This encoding technique allows for the acceleration of operations on the encoded polynomials using FHEEFL.

**Fig. 3:**
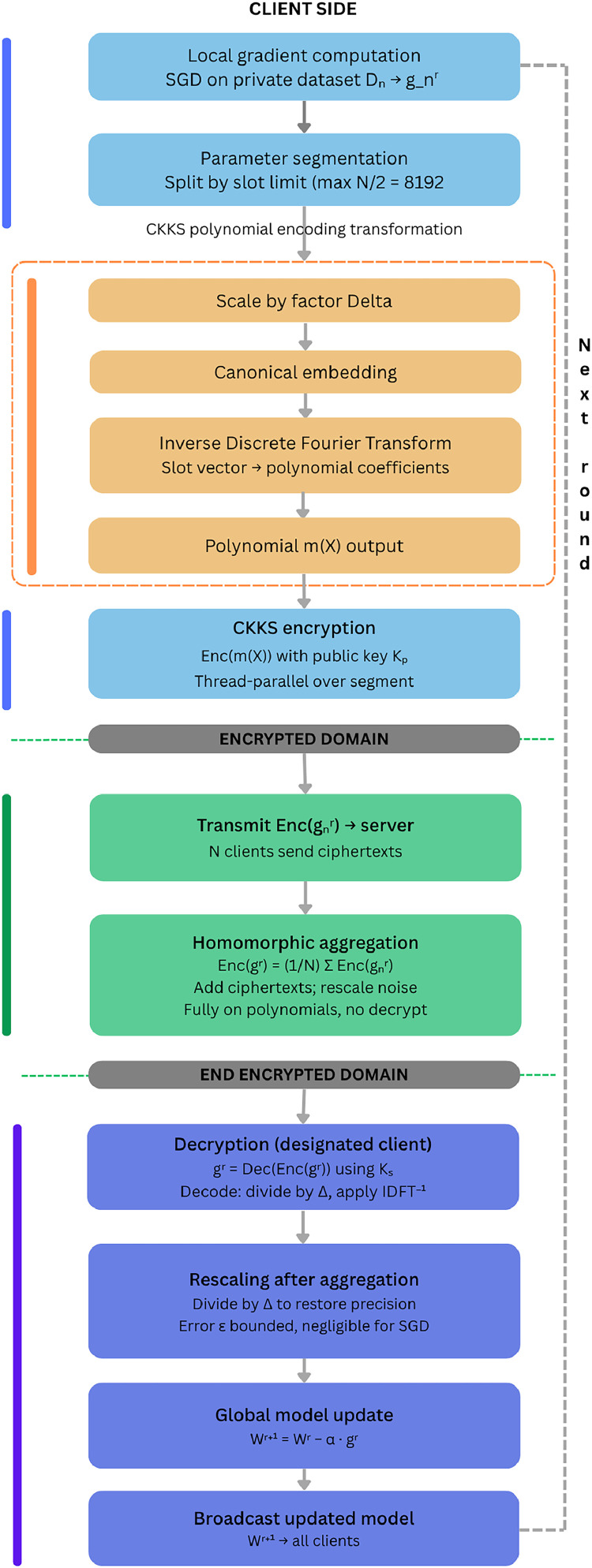
CKKS based Encryption Pipeline for secure aggregation.

Fig. 3 shows the encryption pipeline using Cheon Kim technique along with the training flow. Initially data generated at local devices will be encoded in plaintexts. Plaintexts are floating-point numbers in the vector form. In CKKS, plaintexts to polynomials, m(x) conversion takes place. Then public keys used to encrypt these polynomials. Thus, plaintext is changed into a secure as well as operationally competent format. Once the data d is encrypted into a couple of polynomials denoted as C, a number of operations provided by CKKS are performed on it- addition, multiplication and rotation. We have chosen CKKS as it inherently supports floating-point arithmetic which is much needed in the federated IIoT environment. A function by f is constructed using a constitution of homomorphic operations. This function f is used to decrypt ciphertext C’ = f(C) by using a secret key which outputs P’ = f(P). Similarly, if we decode it, we receive d = f(d). Polynomial encoding transformation is also depicted in encryption pipeline.

### CKKS - components

• Lattice-Based Cryptography. Multiple basis vectors are utilized in random linear combinations to construct Lattices. In our methodology, it serves as the basis of hard problems- closest vector problems.
• Rings. This is a mechanism where addition and multiplication are treated as binary operations and combined in a set. It is used for encrypting plaintext.
• Fields. The field holds all characteristics of the Ring mechanism with some additional properties. This special addition covers inverses for every non-zero element. Furthermore, multiplication in the field is in commutative nature which makes encryption a more robust form. So, Field can be referred to as a special form of Ring.
• SIMD Encoding. SIMD encoding enables us to process multiple data points located at different locations in a federated environment concurrently in a single stroke operation, which will be more helpful in resource constrained IIoT scenarios.
• Cyclotomic Polynomials. It is used to define the ring structure, so that ring operations are carried out efficiently in both encryption and decryption rounds.
• Ring Isomorphisms. It plays a vital role when we transform vector data between different algebraic structures without altering the underlying relationships, thereby adapting the data for cryptographic needs.

In our method, conversion of data space D to the cipher space C using a ring homomorphism employs a map:

**σ**: D ⇒C as shown below:

(4) for∀a,b∈D,σ(a)+σ(b)=σ(a+b)

(5) σ(a)*σ(b)=σ(a*b)

Alternatively, given two data values d1 and d2, a ring homomorphism encryption function *Enc* (⋅) satisfies the properties as indicated below,

(6) Enc(d1)+Enc(d2)=Enc(d1+d2)

(7) Enc(d1)*Enc(d2)=Enc(d1*d2)

As FHEEFL can employ both multiplicative and additive homomorphism, it is justified that it is Fully Homomorphic Encryption (FHE).

The process of integrating FHE into FL involves several key steps, as implemented in the provided codebase:

1. Global Context and Key Generation

The process begins with the generation of a global encryption context using TenSEAL. A full context is created with the CKKS scheme, specifying a degree of polynomial modulus as 32,768 and [60, 40, 40, 60] as bit sizes of coefficient modulus. This context includes both a public encryption key and a secret decryption key which is always secured and not known to the server to ensure that it cannot decrypt the data.

2. Public Key Sharing with Clients

The public context is shared with all clients, as implemented. Each client loads the public context and uses it to encrypt their model weights. This ensures that all clients use the same encryption parameters, enabling secure and compatible computations.

3. Client-Side Model Training and Encryption

Each client trains a local multi-layer perceptron (MLP) model using Pytorch. The model architecture consists of dense layers with layer normalization and L2 regularization. After training, the model weights are flattened and encrypted using the CKKS scheme with the public context. The encrypted weights are serialized and saved to a file, and plaintext weights are saved for verification.

4. Server-Side Aggregation

The server loads the public context and the encrypted weights from all clients. It performs homomorphic averaging by adding the encrypted weight vectors and scaling by the inverse of the number of clients. This operation is performed directly on the encrypted data, ensuring that the server never accesses the plaintext weights. Model parameters are aggregation can also be done sequentially in segments to accommodate the full architecture.

5. Client-Side Decryption

A designated client with access to the secret key, loads the full context and the averaged encrypted weights. The client decrypts the weights, reshapes them to match the model architecture, and sets them into a new MLP model. The decrypted weights are verified against the average of the original plaintext weights using metrics like cosine similarity, Mean Absolute Error (MAE)and Root Mean Square Error (RMSE).

The overall process follows steps shown in Algorithm 2 below:

**Algorithm 2:** FHEEFL.

**Input:** Local Dataset D_n, K_p_, K_s_, W^0^
**Output:** Updates to Global model
1. Initialization
2. Set CKKS parameters
3. Generate public key K_p_
4. Generate secret key K_s_
5. Share K_p_ with client n ą {1, ……, N}
6. Initialize global model W^0^
7. For each global round r = 1 to R;
8. Client – side:
9 Each client n ą {1, ……, N}
10. Receive global model W^r^
11. Train on local dataset D_n
12. Compute gradient g_n^r^
13. Partition g_n^r^ into CKKS slots
14. Encrypt each using K_p_
15. Send Enc(g_n^r^) to sever
16. Server – side:
17. Compute Enc(g^r^) = (1/N) ∑ Enc g_n^r^
18. Client – side
19. Designated client:
20. Decrypt g^r^ using K_s_
21. Update model
22. W ^(r+1)^ = W^r^ - ă * g^r^
23. Broadcast updated model W ^(r+1)^

Table 1 shows the notations used throughout the description.

**Table 1:** Notations used.

Notation	Description
N	The number of the clients
$W_{G}^{0}$	Global model weight
$g_{n}^{r+1}$	Gradient update computed by client n during round r + 1
α	Learning rate
D, |D|	Dataset for training, Dataset size
λ	Regularization Parameter
SGD	Stochastic Gradient Descent
$K_{p,\;}K_{s}$	Public key and secret key

Table 2 shows the Configuration Parameters and CKKS Parameters.

**Table 2:** Configuration parameters and CKKS parameters.

Operating System	Windows 11 Pro (64-bit)
Network	35=> 256=>256=>15
CPU	Intel Core i7–12700H, 4.7 GHz
RAM	32GB
Python	Version 3.12
PyTorch	Version 2.1.0
Model	MLP
Neural network parameters	MLP architecture with 128, 64 hidden units
Scheme	CKKS
CKKS Polynomial Modulus Degree	32,768
Global scale	2^40^
Coefficient modulus bits	[60, 40, 40, 60]
Encryption Library	TenSEAL v0.3.15
Dataset	Edge-IIoT
Evaluation metrics	Plaintext size vs Encrypted Text size, Encryption Time, Decryption Time, Aggregation Time, Memory Usage, CPU Usage

If we consider the concurrency introduced in proposed work, it occurs two times. First, when the gradients are encrypted after local training and second at the time of decryption. To reduce the computational burden linked to CKKS encryption, a parallelization approach using threads was implemented on the client side. Given the slot constraints of CKKS, the 78,863 parameters of the MLP model are divided into several parts. Each part is encrypted simultaneously with separate threads, enabling concurrent ciphertext creation. This threading approach greatly decreases encryption time compared to a fully sequential process while maintaining cryptographic accuracy. The server then conducts homomorphic aggregation on the encrypted parts without needing any secret keys. The combined ciphertext is decrypted only by a specific client with the secret key. In our experimentation all the local clients send the gradients to the server. The server functions entirely within the encrypted domain and does not engage in decryption, thus preventing key exposure and enhancing the security framework for IIoT applications.

## Method validation

**Fig. 4:**
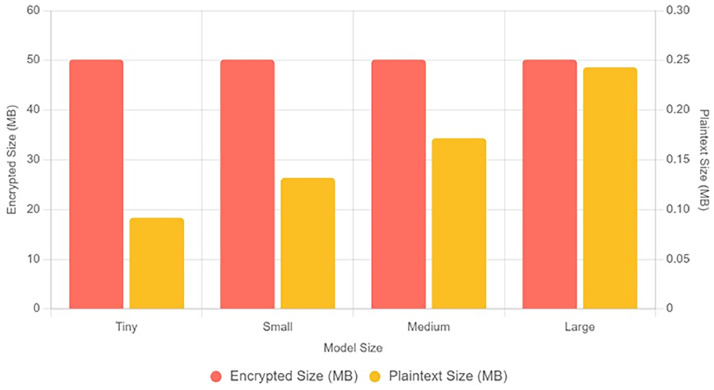
Comparison of encrypted size and plaintext size across different model sizes (Tiny, Small, Medium, and Large).

**Fig. 5:**
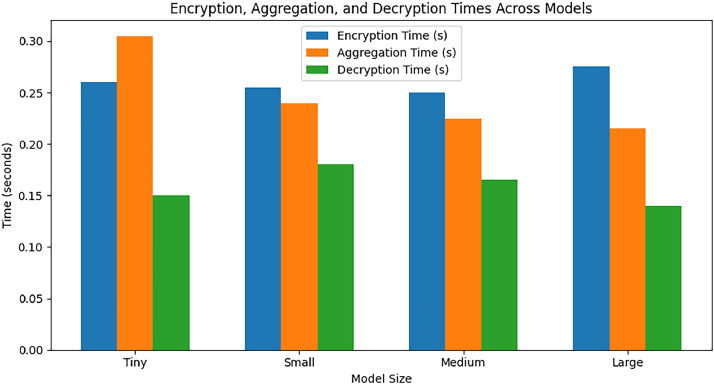
Performance evaluation of encryption, aggregation, and decryption processes across different model scales.

The proposed method FHEEFL has applied on Edge-IIoTSet [20], which is created by adopting various IoT devices and actuators. The method is implemented using the CKKS scheme and the TenSEAL library, detailing the steps from context generation to model aggregation and decryption, along with limitations and performance metrics. In this implementation, we use CKKS with a polynomial modulus degree of 32,768 and coefficient modulus bit sizes of [60, 40, 40, 60], balancing computational accuracy and efficiency. Slots used are N/2 i.e. 32,768 / 2 = 16,384. Model parameters are 78,863. Fig. 4 shows the plot of Plain text vs encrypted text for 4 model variations Tiny, Small, Medium and Large. It can be observed that with increase in plaintext size, method is succeeded to limit the encrypted data size up to 50 Mb. Fig. 5 shows the comparative plot for encryption time, decryption time and aggregation time for 4 model sizes. It can be said that aggregation time gets lesser with increase in model size. The selected MLP architecture comprising 78,863 parameters reflects a practical model size for Edge-IIoTset analytics tasks such as fault diagnosis and predictive maintenance, where lightweight to moderate-capacity networks are typically preferred over very deep architectures due to resource and latency constraints.

If we conceptually compare the FHE approach with existing privacy preserving approaches like PHE (Partial Homomorphic Encryption), SMPC (Secure Multi party computation) and DP (Differential privacy), it is obvious that FHE incurs additional computational and communication overhead. But the prime motive of this work is to reinforce privacy guarantees rather than optimize computational speed. Table 3 covers the conceptual comparison of mentioned four approaches. Unlike other approaches mentioned above that are archetypally restricted to additive operations and there is possibility of partial exposure of plaintext during aggregation process, the FHEEFL framework employs both addition and multiplication directly over ciphered data. This removes intermediate decryption steps and eliminates the risk of gradient leakage. Consequently, although FHE has additional overhead, it furnishes a more comprehensive cryptographic security model, which is utmost important for privacy-sensitive IIoT environments.

**Table 3:** Conceptual comparison of FHE, PHE, SMPC and DP.

Feature	SMPC-FL	DP-FL	PHE-FL	FHEEFL
Encryption used?	No (Secret Sharing)	No (change in weights)	Yes	Yes
Encryption Type	Masking	Noise based	Additive	Additive + Multiplicative
Decryption style	Share reconstruction	None	Final decryption	Final decryption
Who performs it?	Multiple parties jointly	NA	Owner (client node)	Owner (client node)
Intermediate Plaintext Exposure?	No (unless collusion)	Yes (noisy plaintext)	Possible if protocol extended	No
Accuracy impact	Minimal	Moderate	Minimal	Minimal
Computation Cost	Low–Moderate	Low	Moderate	Higher
Communication Cost	High (multi-round)	Low	Moderate	Moderate
Trust Assumption	Honest majority	Trusted server	Semi-trusted	No trust in server
Security Level	Update hiding	Statistical privacy	Partial encryption	Fully encrypted computation

In real time IIoT deployments, communication overhead and latency are critical concerns. If we compare with plain FL framework, CKKS-based encryption causes increased weights update size due to expanded ciphertext. The reason behind is polynomial encoding and modulus representation. This results in higher per-round communication charge. Nevertheless, due to ciphertext packing in CKKS, multiple parameters are encoded in a single ciphertext which partially reduces the bandwidth growth. Counter to the SMPC approach which requires several interactive rounds, FHEEFL achieves non-interactive encrypted aggregation at the server, dropping synchronization delays. Even if homomorphic operations incur computational latency, the trade-off affords stronger cryptographic privacy guarantees suitable for high-security industrial environments. To reduce communication cost further, compression techniques can be employed.

**Fig. 6:**
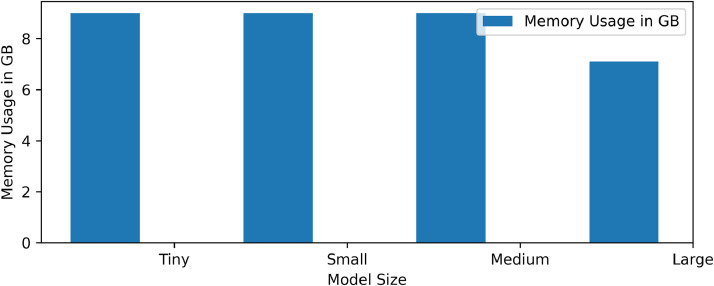
Comparison of memory usage for varying model sizes.

Fig. 6 shows time breakdown among encryption time, decryption time and aggregation time for all models. Time taken for decryption is least followed by aggregation time.

**Fig. 7:**
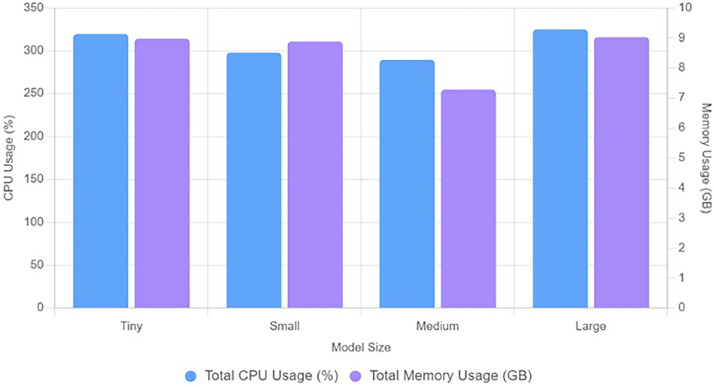
Analysis of total usage of CPU in percentage and memory in GBs.

**Fig. 8:**
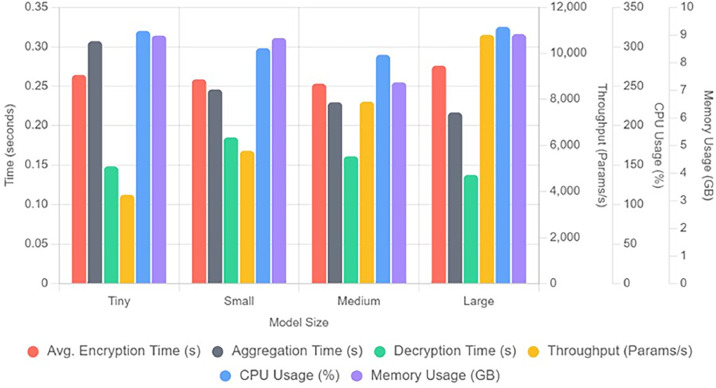
Overall performance analysis.

**Fig. 9:**
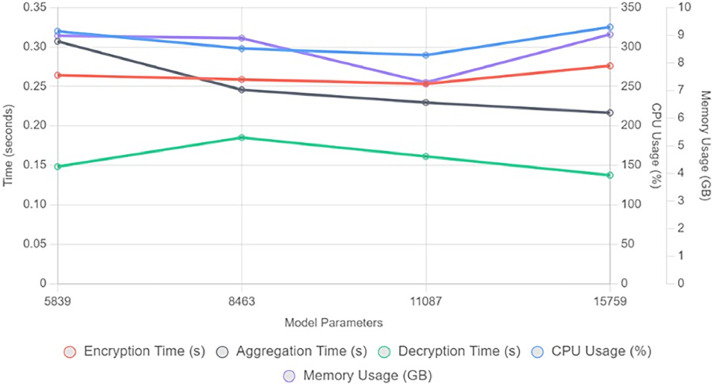
Performance scaling among different numbers of parameters.

Fig. 7 gives the analysis of total usage of CPU in percentage and memory in GBs. We can say that a medium size model is more economical with respect to CPU usage and memory usage. Fig. 8 shows overall performance analysis in a single graph. The new parameter added is a throughput and we can observe that large model use can give maximum throughput around 10,200 parameters. Fig. 9 shows performance scaling among different numbers of parameters- 5839, 8463, 11,087, 15,759.

**Fig. 10:**
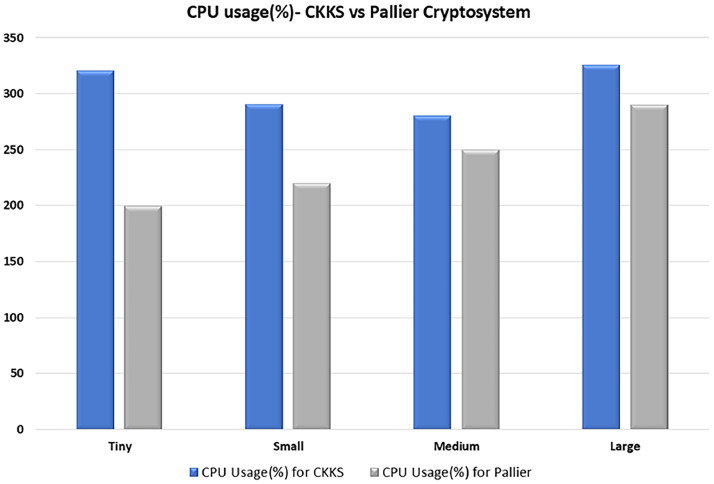
CPU usage comparison across CKKS and Pallier Cryptosystem for varying model sizes.

**Fig. 11:**
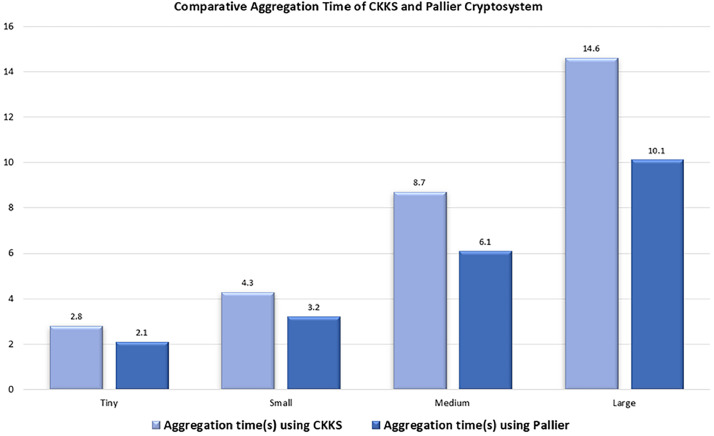
Aggregation time comparison across CKKS and Pallier Cryptosystem for varying model sizes.

**Fig. 12:**
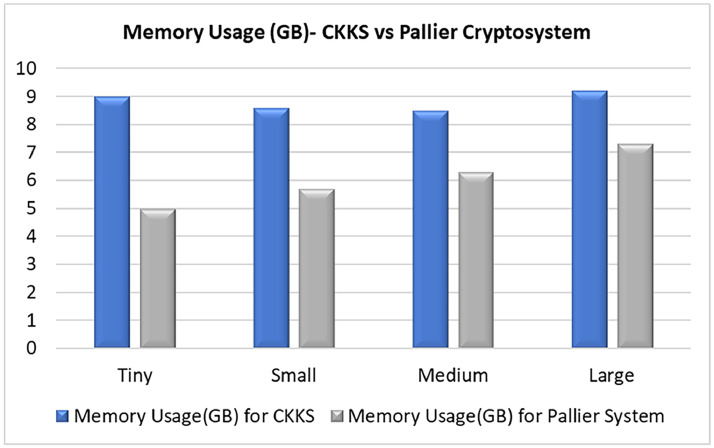
Memory usage comparison across CKKS and Pallier Cryptosystem for varying model sizes.

To get the clarity on performance of proposed work, comparison is done with a Paillier cryptosystem. The term Paillier refers to a prominent Partial Homomorphic Encryption (PHE) cryptosystem, first proposed by Pascal Paillier in 1999 [21]. Paillier is considered a semi-homomorphic or partial encryption scheme because it mainly facilitates additive operations on encrypted data, unlike Fully Homomorphic Encryption (FHE), which allows for arbitrary computations. Detail discussion of Paillier cryptosystem is omitted for brevity. Performance comparison is done in terms of Aggregation Time, CPU Usage (%) and Memory Usage (GB) and results are plotted in Fig. 10, Fig. 11, Fig. 12.

In real time deployment scenarios, it is crucial to account for the computational demands introduced by CKKS-based fully homomorphic encryption, especially in edge environments with limited resources. Tasks such as encoding, encrypting, multiplying ciphertexts, and rescaling considerably increase CPU usage and memory requirements compared to plaintext federated learning. This can result in greater latency for each communication round, which may impact applications that are sensitive to timing. Nonetheless, the proposed FHEEFL framework addresses this issue by employing a lightweight model design, processing ciphertexts in segments, and utilizing thread-based parallel encryption on the client side. Additionally, in numerous IIoT scenarios—like intrusion detection, anomaly monitoring, and periodic analytics—immediate real-time responses are not essential, allowing for a practical balance between privacy and latency. For scenarios that are extremely sensitive to latency, deployment can be enhanced through hardware acceleration, adaptive client participation, or hybrid encryption methods to maintain both efficiency and security.

### Limitations and future scope

Although the proposed FHEEFL framework meaningfully augments privacy through CKKS-based fully homomorphic encryption, some practical boundaries should be acknowledged. First, the CKKS scheme uses a polynomial modulus degree of 32,768, offers 16,384 encoding slots (N/2), restricting the number of parameters that can be packed into a single ciphertext. To lodge the 35–256–256–15 MLP architecture with 78,863 parameters, due to the CKKS slot limitation, model parameters can be encrypted and aggregated sequentially in segments to accommodate the full 78,863-parameter architecture. While this approach enables secure training of moderate-sized networks, it increases aggregation latency proportionally to the number of parameter segments processed. Consequently, scaling to very deep architectures commonly used in large-scale industrial analytics would significantly increase computational and communication overhead. Therefore, the proposed approach is particularly apt for lightweight to medium-scale models generally deployed in Edge-IIoT environments. We would like to suggest that the parameter limit can be overcome by employing ciphertext partitioning, block wise encryption or model compression.

Second, homomorphic encryption operations—including encoding, encryption, ciphertext multiplication, rescaling, and aggregation—are computationally intensive compared to plaintext federated learning. This results in increased CPU utilization and stretched aggregation latency per communication round. In real-time IIoT scenarios with strict timing constraints, such overhead may introduce response delays. Thus, FHEEFL is particularly well-suited for privacy-critical analytics tasks such as fault diagnosis, anomaly monitoring and predictive maintenance, where moderate latency trade-offs are acceptable.

In conclusion, the computational cost of FHE may affect energy consumption on resource-constrained edge devices. Future work will explore optimization strategies including model gradient compression, communication scheduling, and hardware-aided acceleration to progress real-time feasibility and energy efficiency.

## Ethics statements

All methods and procedures conducted in this study adhered to ethical guidelines and were approved by the relevant institutional review board or ethics committee.

## CRediT author statement

Shraddha Subhedar: Proposed Algorithm, Formal analysis, Visualization, Writing – original draft, Writing – review & editing. Dr. Deepa Parasar: Review, Project administration, Supervision.

## Supplementary material *and/or* additional information [optional]

None.

## Declaration of competing interest

The authors declare that they have no known competing financial interests or personal relationships that could have appeared to influence the work reported in this paper.

## Data Availability

Data will be made available on request.
